# Case of refractory delirious mania responsive to lithium

**DOI:** 10.1192/bjo.2021.957

**Published:** 2021-06-28

**Authors:** Manuel Pereira Herrera, Aphrodite Marta Zimmerman

**Affiliations:** Department of Internal Medicine, Overlook Medical Center, New Jersey, USA; Department of Psychiatry, Overlook Medical Center, New Jersey, USA

**Keywords:** Bipolar affective disorders, delirious mania, neuroleptic malignant syndrome, catatonia, lithium

## Abstract

Delirious mania is an uncommon condition that is challenging to diagnose and treat. More often seen in patients with a history of bipolar disorder, it frequently presents with prominent catatonic features and overlaps with other diseases in the catatonic spectrum, such as neuroleptic malignant syndrome, serotonin syndrome and malignant catatonia. We present a case of delirious mania refractory to high doses of lorazepam, several antipsychotics and valproic acid, which responded dramatically to therapy with lithium after 26 days of minimal improvement with the other medications. The pathophysiology of delirious mania is reviewed, and the unique actions of lithium are discussed, highlighting possible reasons as to why lithium might offer advantages in the treatment of this disease.

Delirious mania is an uncommon neuropsychiatric condition that is challenging to diagnose and treat. There are no major studies or randomised trials concerning delirious mania, and the DSM-5 does not include it in its listing of psychiatric conditions. From case reports, it can be characterised as involving signs and symptoms of mania, delirium and, occasionally, catatonia.^1–4^ In 2001, Fink and Taylor categorised delirious mania as belonging to the catatonic spectrum, a collection of related syndromes including catatonia, serotonin syndrome and neuroleptic malignant syndrome (NMS).^5^ The prompt recognition of delirious mania is essential as, without adequate treatment, it can decompensate into a malignant form with haemodynamic instability, hyperthermia, rhabdomyolysis, acute kidney injury and cardiovascular collapse, similar to NMS.^6^

The pathophysiology of delirious mania is not completely understood, but, similar to catatonia, it likely involves dysregulation of gamma-aminobutyric acid (GABA)ergic and glutamatergic networks, resulting in a hyperdopaminergic state that leads to an excessive sympathetic output.^7,8^ Studies have also mentioned the role of neuroinflammation and acute insults to the corpus callosum as possible precipitants.^4,9^

Recommended treatments include high doses of benzodiazepines, second-generation antipsychotics, mood stabilisers and electroconvulsive therapy (ECT) in severe cases.^1–3^ We present a case of delirious mania refractory to benzodiazepines, several second-generation antipsychotics and valproic acid (VPA), which achieved marked improvement 3 days after initiation of therapy with lithium.

## Case presentation

A 33-year-old, single, unemployed homeless man was transferred to our hospital from a drug rehabilitation facility where he had voluntarily enrolled 10 days prior. He had been somewhat agitated and disorganised earlier during his stay, and had progressively deteriorated since that time; his attempts to consume gloves and soap prompted his transfer. He had a history of bipolar disorder, obsessive-compulsive disorder, several episodes of mild traumatic brain injury, and hypertension. Intolerance to haloperidol was reported. His substance use included prescription opioids (unknown kind and quantity), benzodiazepines (reported use of 4–14 mg alprazolam daily) and regular cannabis use (unknown quantity), and he was an active tobacco smoker of approximately 20 cigarettes per day for the past 10 years. He had no history of alcohol misuse. His family indicated he had been depressed and functioning poorly in the community before recent events. His medications at the rehabilitation centre included quetiapine (300 mg nightly), carbamazepine (200 mg daily), benztropine (2 mg twice daily), gabapentin (400 mg three times daily), fluoxetine (40 mg daily), buprenorphine (8 mg daily) to prevent opioid withdrawal, chlordiazepoxide (25 mg as needed), hydroxyzine (50 mg as needed) and oral clonidine (0.1 mg as needed).

The patient, who was extremely agitated and combative, arrived with a police escort and was placed under four-point restraints. He was well developed and properly groomed. He was afebrile, his blood pressure was 131/83 mmHg, his heart rate was 80 beats/min, his respiratory rate was 20 breaths/min and his peripheral oxygen saturation was 96%. He had pressured speech, paranoid ideation and was disoriented to place and situation. He received intravenous diazepam (three doses of 10 mg and two doses of 5 mg), nine doses of 2 mg intravenous lorazepam, 50 mg oral quetiapine and 20 mg ziprasidone, all of which had no effect on his agitation. He was finally sedated with a dexmedetomidine infusion and transferred to the intensive care unit.

Initial laboratory tests showed mild anaemia (haemoglobin 11.8 g/dL), normal electrolytes, mild transaminitis (aspartate transaminase 60 U/L and alanine transaminase 83 U/L) and elevated creatinine kinase (1215 IU/L). His urine drug screen was only positive for benzodiazepines, which he had received in the emergency room before his urine sample was taken and previously had received at the rehabilitation facility. His chest X-ray was normal, and his electrocardiogram showed a mildly prolonged corrected QT interval of 490. His head computed tomography and brain magnetic resonance imaging scans were normal.

He was started on intravenous fluids, intravenous thiamine, and folic acid. On day 2, he was still sedated under the dexmedetomidine infusion, but would awaken and begin babbling incoherently, yelling profanities and violently thrashing around in his restraints. The working diagnosis was acute withdrawal from an unknown substance. He was started on 4 mg intravenous lorazepam every 6 h, with an additional 3 mg every 6 h as needed, along with 50 mg intramuscular chlorpromazine every 6 h as needed for severe agitation, in light of his recorded haloperidol intolerance. There was also suspicion of mania, given his history of bipolar disorder, so he was started on 50 mg quetiapine three times daily and 500 mg intravenous valproate twice daily. He continued to receive 4 mg buprenorphine twice daily for opiate dependence. His mental status was unchanged over the course of several days, despite multiple doses of intravenous lorazepam, upward of 20 mg daily, in addition to multiple doses of intramuscular chlorpromazine. His intravenous valproate was increased to 750 mg twice daily.

On day 6, he was observed to have oedema of his right deltoid, a fever of 39.4°C and tachycardia (120 beats/min). He was still incoherent, paranoid and aggressive, but he had a normal muscle tone. His creatinine kinase was elevated at 2438 IU/L; his antipsychotics were held, owing to possible incipient NMS. However, magnetic resonance imaging of his right arm showed a deltoid abscess, likely secondary to his multiple intramuscular chlorpromazine injections, so intravenous vancomycin was started. Following initiation of antibiotics, he remained afebrile and appeared to have slight improvement in his confusion. By the next day, his creatinine kinase had decreased to 1542 IU/L.

On day 8, he coursed with urinary retention, requiring a Foley catheter placement. Neurology was consulted and a lumbar puncture was done. Cerebrospinal fluid studies, including glucose, protein, cell count, paraneoplastic antibodies, Gram staining, cultures and assays for bacterial and viral encephalitis were all negative. An electroencephalogram showed bi-hemispheric irregular slowing, which was deemed to be secondary to his medications. On day 9, his behaviour alternated between periods of agitation/aggression and lethargy/unresponsiveness. Risperidone was added and titrated up to 2 mg twice daily, but he experienced worsening lethargy and decreased oral intake, and he persisted with unintelligible speech. A repeat electroencephalogram was normal. Risperidone was discontinued, and he was maintained on quetiapine, now at 400 mg twice daily, and 1500 mg valproate daily. His valproate blood level was 57 μg/mL. By day 16, the working diagnosis was delirious mania, and he was transferred out of the intensive care unit.

He had minimal improvement on the medical floors. On day 21, discussions with the family about pursuing court-ordered ECT were revisited; prior attempts to discuss ECT had been unsuccessful because of disagreement among family members about this option. On day 23, he was started on 300 mg lithium three times daily, and his quetiapine was changed to 200 mg daily plus 600 mg nightly. The family agreed to begin the process of a court order for ECT.

Remarkably, by day 26, 3 days after initiation of lithium, he was euthymic, talkative, fully oriented and asking questions about his medications. This represented a dramatic change from his overall course up to this point, since he had remained essentially delirious, alternating between agitation and unresponsiveness and with unintelligible speech for over 3 weeks. In light of this change, the valproate was deemed ineffective and discontinued. On day 27, the patient was pleasant and cooperative but had limited memory of the past 26 days. He verbalised that he ‘wanted to get his life together’ and was voluntarily transferred to the psychiatric unit for further treatment. At this point, his medications were 200 mg quetiapine daily plus 600 mg nightly, 300 mg lithium three times daily, 2 mg buprenorphine twice daily and 0.1 mg clonidine twice daily. He was discharged a few days later and was lost to follow-up. See Table 1 for a full summary of the clinical course.

**Table 1:** Summary of clinical course

Day	Treatment	Clinical condition
Day 0, admission	Intravenous lorazepam total of 40 mg, ziprasidone 20 mg, quetiapine 50 mg and dexmedetomidine infusion	Violently agitated, then sedated after dexmedetomidine
Days 1 and 2	Continued dexmedetomidine infusion, intravenous lorazepam 4 g every 6 h, intramuscular chlorpromazine 50 mg every 6 h as needed, quetiapine 50 mg three times daily, intravenous valproate 500 mg twice daily, buprenorphine 4 mg twice daily, clonidine 0.1 mg daily	Delirious, dysarthric, incoherent and agitated
Days 3–6	Increased intravenous valproate to 750 mg twice daily, added intravenous vancomycin, stopped all antipsychotics, stopped dexmedetomidine	Mental status unchanged, now febrile and tachycardic, with increasing creatinine kinase
Days 7 and 8	Continued intravenous valproate 750 mg twice daily	Fever resolved, creatinine kinase decreased, developed urinary retention, mental status alternating between agitation and unresponsiveness
Days 9 and 10	Added risperidone and titrated to 2 mg twice daily	Developed worsening lethargy after increasing risperidone, continued delirium with unintelligible speech
Days 11–22	Risperidone stopped, added quetiapine and slowly titrated to 400 mg twice daily, continued intravenous valproate 750 mg twice daily	Lethargy slightly improved, still delirious and incoherent, with minimal change
Day 23	Started lithium 300 mg three times daily, increased quetiapine to 200 mg daily plus 600 mg nightly	Still delirious, mostly lethargic, incoherent and dysarthric
Days 24–26	Stopped valproate because of improvement with lithium	Gradual improvement until euthymic, conversant and fully oriented
Day 27	Continued quetiapine 200 mg daily plus 600 mg nightly, lithium 300 mg three times daily, buprenorphine 2 mg twice daily, clonidine 0.1 mg twice daily	Remained pleasant, cooperative and oriented, with limited memory of the past 26 days

### Consent

Written informed consent was obtained from the power of attorney (including healthcare and mental healthcare issues) of the patient for the purpose of this case report.

## Discussion

### Diagnosis

The presence of mania and delirium with or without catatonia should suggest a diagnosis of delirious mania.^8^ Briefly, delirium is a syndrome that presents with acute psychomotor hypoactivity or hyperactivity and confusion (i.e. reduced clarity or awareness of the environment), with reduced ability to sustain focus or shift attention. Delusions and hallucinations are common, and symptoms classically wax and wane. Mania involves the presence of abnormally and persistently elevated, expansive or irritable mood, along with excessive goal-directed activity or energy, pressured speech, distractibility, racing thoughts and grandiosity persisting for at least 1 week. Symptoms usually develop within hours to days. Delirious mania can appear in patients without a history of bipolar disorder.^10^ Drug intoxication/withdrawal, electrolyte abnormalities, and hormonal, infectious, pharmacological and structural causes need to be ruled out. In addition, encephalitis should always be considered in the differential diagnosis of delirious mania.^4,8^ There is a high incidence of catatonic findings in delirious mania, and the term delirious catatonia has been suggested as more illustrative of the actual clinical syndrome.^1,2,8^ Both catatonia and delirious mania may worsen to NMS after the administration of first-generation antipsychotics.^11^ Signs of delirium are more abundant in the nonmalignant delirious mania.^1^ Lack of response to benzodiazepines may indicate progression to malignant delirious mania.^1^

### Pathophysiology

Given the overlap between delirious mania and catatonia, it is relevant to discuss the pathophysiology of catatonia, which seems to involve neural pathway dysfunction rather than site-specific dysfunction.^10^ The behavioural/affective symptoms in catatonia are possibly related to problems in the orbitofrontal cortex, which is involved in social adjustment and emotional regulation, and the motor symptoms are likely related to dysfunction in the medial prefrontal cortex, which is involved in monitoring motor tasks.^12^ Given that patients with catatonia tend to respond very favourably to benzodiazepines,^1^ dysfunction in the cortical GABA circuitry likely plays an important role. Single-photon emission computed tomography studies have shown a decrease in the density of GABA-A receptors in the primary motor cortex of patients with catatonia versus controls,^13^ and other studies have also shown downregulation of these receptors in patients who are catatonic.^14^

Hyperfunctioning glutamatergic networks in cortical regions could be a primary mechanism explaining some symptoms of catatonia.^15^ This might account for the efficacy of N-methyl-D-aspartate (NMDA) antagonists such as amantadine or memantine in catatonia, and it could also explain the similarity of symptoms of NMDA receptor encephalitis with delirious mania and malignant catatonia.^16^ Moreover, topiramate, a glutamate antagonist, has been beneficial in catatonia refractory to benzodiazepines.^17^

Neuroinflammation has been associated with delirious mania.^4,9^ Restrepo-Martinez et al found symptoms of delirious mania in 22.7% of 79 patients with NMDA receptor encephalitis.^18^ Delirious mania has also been reported in autoimmune encephalitis involving anti-glutamic acid decarboxylase-65 (anti-GAD65) antibodies.^9^ Recently, Bellani et al reported a case of delirious mania associated with mild encephalitis with reversible splenial lesion, a rare condition that presents with a single lesion in the midline of the splenium of the corpus callosum, of unknown aetiology but possibly caused by cytotoxic oedema.^4^ Studies have shown structural and diffusion-weighted imaging abnormalities in the volume and microstructure of the corpus callosum in patients with bipolar disorder,^19^ pointing to impaired cerebral interhemispheric communication as a contributor to the symptoms observed in this patient population even when they are euthymic.^20^ Additional acute compromise of the corpus callosum in these patients could then precipitate a delirious mania episode.

### Relevance of lithium for delirious mania

Lithium has a variety of neuroprotective, immunomodulatory and anti-inflammatory effects beyond those seen in any other psychotropic agent.^21^ Lithium causes direct inhibition of glycogen synthase kinase 3 (GSK-3),^22^ which is thought to be important in its mood-stabilising and neuroprotective effects. GSK-3 dysfunction has been linked to the pathophysiology of mood disorders, schizophrenia, Alzheimer's disease and metabolic conditions like diabetes.^23^ Lithium also increases the levels of brain-derived neurotrophic factor (BDNF), glial cell-derived neurotrophic factor (GDNF) and vascular endothelial growth factor (VEGF).^24^ In addition, lithium has been shown to increase levels of the antiapoptotic protein B-cell lymphoma 2 (Bcl-2), preventing the activation of proapoptotic caspases and modulating the excitotoxicity caused by the NMDA receptor.^24^

Ferensztajn-Rochowiak et al showed that patients with bipolar disorder who are not treated with lithium but are on anticonvulsants, including VPA, had a higher number of very small embryonic-like stem cells; higher levels of the pluripotency markers octamer-binding transcription factor 4 (Oct-4), sex determining region Y-box 2 (Sox2) and Nanog homeobox (Nanog); and higher levels of the glial cell markers glial fibrillary acidic protein (GFAP), oligodendrocyte transcription factor 1 (Olig1) and oligodendrocyte transcription factor 2 (Olig2).^25^ Increased mobilisation of very small embryonic-like stem cells into the peripheral blood occurs in the presence of systemic inflammation and has been suggested as a marker of bipolar disease progression.^25^ The glial cell markers Olig1 and Olig2 reflect activation of oligodendrocyte differentiation and maturation, as well as repair processes of demyelinated lesions in the adult central nervous system.^25^ Higher levels of GFAP have been found in the frontal cortex of patients with bipolar disorder.^25^ All of these markers were decreased only in the healthy controls and in the patients receiving lithium, and they point to lithium's role in protecting against cell damage from inflammation.^25^

Chronic treatment with lithium has been shown to be protective against oxidative stress.^22^ Increased oxidative stress has been associated with manic episodes in bipolar disorder; several reactive oxygen species were found to be elevated in unmedicated patients with mania compared with patients receiving lithium and healthy controls.^26^ Lai et al demonstrated that the cytoprotective effect of lithium and VPA against oxidative stress, which causes release of cytochrome *c* and activation of caspases, is seen after 7 days or chronic treatment but not after 1 day.^27^ Li and El-Mallahk found that acute pretreatment with lithium, but not with VPA, decreased caspase-3 activation in neuroblastoma cells,^28^ but this was not replicated by Lai et al, who showed that chronic lithium or valproate pretreatment abrogated the activation of caspase-3 in these cells.^27^ Interestingly, neither agent protected glial cells against these insults, suggesting that the action of VPA and lithium may be cell specific.^27^ Chronic treatment with lithium (>7 days) was also shown to decrease phosphorylated cyclic adenosine monophosphate responsive element binding protein (phospho-CREB), but no effects on phospho-CREB were found after acute treatment with lithium or acute or chronic treatment with VPA.^29^

In contrast to VPA, lithium differentially affects a variety of intracellular second-messenger systems, including cyclic adenosine monophosphate (cAMP) and inositol.^30^ Lithium also affects neuropeptide Y levels and decreases calcium influx in the NMDA receptor.^30^ Studies have also found that chronic treatment with lithium, but not with VPA, increases the brain concentrations of N-acetyl-aspartate (NAA), which is present in high levels in neurons and can be used as a non-specific neuronal marker.^31^ NAA levels are decreased in a variety of neurological and psychiatric conditions, including Alzheimer's disease, schizophrenia, amyotrophic lateral sclerosis, post-traumatic stress disorder, geriatric depression and others.^31^

Beyond its molecular effects, lithium has been associated with greater grey matter density in the cingulate gyrus, superior temporal gyrus, postcentral gyrus, hippocampus/amygdala and insula in patients with bipolar disorder on chronic lithium treatment.^32^ In patients with bipolar depression, chronic lithium treatment was also associated with increased grey matter volumes in the dorsolateral prefrontal cortex, orbitofrontal cortex and other areas.^33^ These areas are likely implicated in the pathophysiology of delirious mania, as mentioned previously. All of these considerations raise the possibility that the neurotrophic/neuroprotective effects of lithium are much broader than VPA and could help explain the dramatic turnaround of our patient following initiation of lithium.

### Which helped, valproate, quetiapine or lithium?

It is not possible to completely separate the effects of these three medications in our patient, but several points are worth highlighting. The valproate level of our patient was 57 μg/mL on day 9, which could be viewed as subtherapeutic. However, because of the patient's lethargy and gait instability, it was felt by the treatment team that the dosing of valproate should not be increased further than 750 mg twice daily. Additionally, the dose of quetiapine was titrated to 400 mg twice daily within approximately 10 days before starting lithium; however, the patient had already been on the lower dose of 150 mg quetiapine daily for about 6 days at the beginning of his hospital stay, with virtually no response. Had our patient been responsive to quetiapine, we should have observed a gradual improvement in the initial 6 days of hospital stay and a much greater improvement with the higher dose in the 11–22 days before starting lithium, but this was not observed. Instead, we had a significant clinical response within only 3 days of starting 300 mg lithium three times daily. Therefore, we suspect that the explanation is that his improvement was a result of the lithium and not the quetiapine.

## Conclusions

Through its anti-inflammatory properties, potent neuroplastic actions and broader range of effects compared with VPA, lithium might be especially well suited to treat delirious mania in combination with benzodiazepines and second-generation antipsychotics. This hypothesis should be examined in future studies.

## Data Availability

Data availability is not applicable to this case report, as the information was obtained from the hospital chart of the patient.
